# Editorial

**Published:** 1854-07

**Authors:** 


### Editorial.

---

### OHIO COLLEGE OF DENTAL SURGERY.

We give below the Charter under which the Ohio College of Dental Surgery was instituted, and would remark that, since the act of incorporation one of the trustees has deceased, viz: J. P. Cornell, Esq. The vacancy has been filled by the appointment of Dr. Edward Taylor.

### AN ACT

*To authorize the establishment of a College of Dental Surgery.*

SEC. 1. *Be it enacted by the General Assembly of the State of Ohio,* That B. P. Aydotte, Robert Buchanan, Dr. Israel M. Dodge, William Johnson, J. P. Cornell, and Calvin Fletcher, of Cincinnati, Dr. G. S. P. Hempstead, of Portsmouth, and Dr. Samuel Martin, of Xenia, and their successors, are hereby constituted and appointed a Board of Trustees, with power to establish a College of Dental Surgery, in the city of Cincinnati, and said Board is hereby declared to be a body corporate and politic, with perpetual succession, and shall be known by the name and style of the Trustees af the Ohio College of Dental Surgery; and the said Board shall have power to acquire, hold and convey property for the endowment of said college, to sue and be sued, contract tients or *strangers rather*, call on us for specimens of our work, and as our brother remarked a few days since, when such an one left our office, "She will not return—we never got a job from such an one yet," and I believe we never did. They always want cheap work, and are no better judges of that which is good, than *Hottentots*. The fact is, we never have and never expect to make a specimen operation as a *catch-penny* for business.

### SUBSCRIBERS TO DENTAL PERIODICALS.

It would be well enough for some who take and read Dental Journals for three or four years, without paying anything; to recollect that the list of subscribers for such works must necessarily be small, and hence the necessity for prompt pay. They should also recollect, that if they take the work from the post office, they are as really subscribers, as if they had written to the publishers and ordered the work. Some appear to receive such works from the publishers when sent them, as a mere complimentary affair. When the fact is, they are supposed to be *able, willing, and honorable* enough to pay for the same; and thus give their aid to advance the profession they practice. Such should also recollect that their own interest is thus subserved, for as the profession is advanced, so will be their standing and influence in society, and also remuneration for their services. The fact is, every man in the profession should rejoice to co-operate in this work.

### BROWN'S DENTAL ADVERTISER.

This is a Quarterly issued by Dr. J. M. Brown, our enterprising Dental Depot *Man*. The Advertiser is gotten up in neat style, and indeed so much so, that some to whom it has been sent, have supposed it to be a regular Periodical devoted to the Profession, and would cost them some two dollars per year. In the first supposition they are certainly correct, for it is the interest of the Profession to know what is for sale, which they may need, and also where they can be procured. This is the object of the Advertiser, and the mere fact that it forms a pretty good sized Periodical, and is so full of instructive, and interesting matter, does not necessarily imply that the Dr. charges anything for it. We believe he sends it, postage paid, to all his subscribers and customers free of charge.

### E. E. JOHNSON'S SUPERIOR TOOTH SOAP.

This article has been prepared by Mr. Johnson according to the suggestion of various Dentists in the East as well as West, and combines with it orris root and gum myrrh with some pleasant aromatics, rendering it a very pleasant article for the mouth. Mr. Johnson's object is to supply the profession with an article of this kind, which they can recommend when anything of the kind is needed for their patients, and we believe that he has come nearer accomplishing this, than any one else.

Dr. J. M. Brown is agent for the sale of this article, and can supply the profession on advantageous terms.

### AMERICAN SOCIETY OF DENTAL SURGEONS.

This, the oldest Dental Association in the United States, holds its annual meeting this year in this City, and is to convene on the first Tuesday of August next, at the Burnet House, at 10 A. M. We presume its regular Sessions will be held in the lecture room of the Ohio College of Dental Surgery, which we are assured will be in readiness by that time.

We hope the profession West, will, as far as possible, attend this meeting. Those who are not members will certainly find it interesting and instructive to attend, as the discussions are always open and the profession invited to *attend*.

### DENTAL RECORDER.

We find that we have neglected to notice that the Recorder is edited solely by Dr. A. Hill, of Norwalk, Conn. Dr. Hill is a chaste and forcible writer, and will maintain, we doubt not, the high character of the Recorder.

In bidding Dr. C. C. Allen adieu as an editor, we are happy to be permitted to congratulate him in his new relation, by which he has *doffed* his *Bachelor cap*.

### BLISTER AND CRITIC.

This is a new monthly Medical Periodical, devoted to the development of Southern Medical literature, and the exposition of the diseases, and physical peculiarities of the Negro race. Edited by H. A. Ramsay, M. D., of Atlanta Georgia. It is gotten up in a neat style, and the first number now before us is filled with original and well written articles. We gladly place it upon our exchange list.

### DENTAL REMOVAL.

Dr. Ludlow of this city, has changed his location to Indianapolis, Indiana. This place is rapidly improvng, and we are told now supports some three or four Dentists. We have no doubt but that the Dr. will find it a pleasant and desirable place to reside, and the Profession of that city receive with pleasure a brother so courteous and gentlemanly as Dr. Ludlow.

### DR. BLANDY'S DENTAL MANUFACTORY.

We would draw attention to the advertisement of Dr. A. Blandy, in the present number of the Register. We should think that with such an assort- of moulds and materials, that Baltimore might be made a most admirable point to carry on such an establishment ; and that some one who had more time than Dr. Blandy to attend to it, might make it a very profitable concern.

### RECEIPTS FOR THE REGISTER.

Dr. J. Scott, Pittsburg, Pa., vol. 7,..$2.00
Dr. G. Watt, Xenia, O., vols 6 & 7,..4.00
Dr. Saml. P. Cutter, Holly Springs. Miss., vol. 7,........................2.00
Dr. Johnson, Indianapolis, Ind., vol. 7,.2.00
Dr. W. W. Granger, Fairmount, Va., vols. 6 and one-half of 7,.............3.00
Dr. H. Judd, Warsaw, Ill, vol 7,....2.00
Dr. H. Munro, Lebanon, Ky.,.........2.00
Dr. S. Hughes, Fairfield, Ind.,........2.00
Dr. G. G. Hildreth. Liberty, Mo.,.....2.00
Dr. D. M. Smith, Canton, Ill.,.........2.00
Dr. M. M. Oldham. Springfield, O.,...3.00
Dr. J. Kegertea. Oshkosh, Wis.,......2.00
Dr. Josiah Ramsay, Springfield, O.,...2.00
Drs. S. Hawkins & Ely, Union, O.,...2.00
Dr. H. McCullom, Augusta, Ky.,.....2.00
Dr. J. C. Ross, Nashville, Tenn., vols. 6 & 7,................................4.00

and be contracted with, plead and be impleaded, defend and be defended, answer and be answered unto, in all courts and places, and in all matters and causes whatsoever; provided that no part of the estate, either real or personal, which said corporation may at any time acquire, shall be employed in the business of banking, or for any other purpose than that designated by this act; and provided also, that the revenues arising from the property which said incorporation shall be permitted to hold, for the purposes above specified, shall not exceed the sum of five thousand dollars per annum.

Sec. 2. That the said incorporation may have a common seal, which may be altered, broken or renewed, at pleasure.

Sec. 3. That the officers of said incorporation shall be a President, Vice President, Register and Treasurer, who shall be elected annually, by said Board of Trustees, at such time and in such manner as the said Board may direct, and shall hold their offices until their successors are chosen.

Sec. 4. That the Trustees of the aforesaid incorporation shall have full power to create and establish such professorships as they may deem necessary for said college, and that they may, at any time, appoint or dismiss all such professors or lecturers as they may think proper; also to make and ordain such by laws, rules and regulations as they may deem necessary for the government and well-being of said college; provided such by-laws, rules and regulations are not inconsistent with the constitution and laws of this State and of the United States; and provided also that no branches of medical science shall be taught except those necessary to dental surgery.

Sec. 5. That all vacancies which may occur from death, resignation or otherwise, in the Board of Trustees of the aforesaid incorporation, shall be filled by the remaining members of said Board.

Sec. 6. That the said Board of Trustees shall have power and are hereby authorized to confer the degree of Dr. of Dental Surgery, and grant diplomas for the same, under the seal of the incorporation; provided that no diploma thus granted shall confer any privilege further than the practice of dental surgery.

Sec. 7. That the said corporation shall be subject to all the regulations and liabilities of an act instituting proceedings against corporations not possessing banking powers, and to provide for the regulation of corporations generally, passed March 7th, one thousand eight huudred and forty-two.

Sec. 8. This act shall take effect from and after its passage.

JOHN M. GALLAGHER,
*Speaker of the House of Representatives.*
DAVID CHAMBERS,
*Speaker of the Senate.*

*January* 21, 1845.

---

PORCELAIN TEETH.

The profession cannot but feel gratified at the competition which is so evidently springing up in the manufacture of teeth.

In our last, we took occasion to notice some specimens of teeth, manufactured by Mr. Armstrong of Philadelphia. It appears that with some, our remarks have led them to suppose that the peculiarity of style, and adaptation which we then noticed and recommended, was strictly an improvement by this gentleman. We do not know that Mr. Armstrong claims to have made this improvement, and indeed we know not who introduced it. Single gum teeth made for sets, should as nearly as possible, when adjusted to the [illegible]e, resemble block teeth, and those requiring the least grinding to make them fit, are certainly very desirable to the Dentist.

Many of Mr. Alcock's teeth possess this peculiar advantage, and indeed their adaptationand naturalness of form are their great recommendations; perhaps there are no teeth, as a general rule, so neatly carved. The teeth made by Morton of Boston, a few of which were sent to this market, were made in sets to resemble blocks, and in this particular answered admirably, but they lacked transparency and life-like coloring.

Teeth made by Messrs. Jones, White and McCurdy, are also some of them thus arranged in sets, and possess these qualities. For strength of body, capability of repeated heatings, transparency and life-like appearance in the mouth, their teeth have no superior. We think the oft-repeated expression "as good as Jones, White & Co.'s," the best commendation. We may take some of these qualities named, and perhaps find them fully equalled by teeth of other manufacterers, but the blending of the whole will be difficult to find in any other. We say this without any disparagement to other gentlemen engaged in the same business, for every Dentist who has used the teeth of Stockton, Alcock, Armstrong, Wardle & Co., McIlhenny & Co., Klein, &c., &c., will at once be ready to say : either supply a far better article than could be procured but a few years since. We presume that there is no Dentist but would be glad to avail himself of a selection from the stock of any of these gentlemen's teeth, and that for special occasions, he would thus be better able to meet the wants of his patients. The fact is, the variety of style is such that no one appears to be able to meet the entire demand.

---

### AWARDS AT THE WORLD'S FAIR ON DENTAL SPECIMENS.

We see from the eastern Journals, that quite a breeze has sprung up on this subject, and had we room we should like to let the Profession West, enjoy some of the rich sport, and be enabled to congratulate the highly honored and lucky *ones.* We feel, indeed, that we cannot for three months to come, keep the Profession *out West*, ignorant of the awards *made and given.* It appears first, that R. T. Reynolds, M. D., of Philadelphia, got a *certificate* for superior workmanship in Mechanical Denistry.

Second, That Norman W. Kingsley of New York, got a *certificate* for imitating discolored spots on block teeth, which appear at times on the natural organs.

Third, That Messrs. Jones, White & McCurdy, of Philadelphia, get a *bronze medal, and certificate for important* improvements in the manufacture of the best artificial teeth.

Fourth, That Messrs. Abby & Son, get a bronze medal for the best metalic foils.

Here is a fine array of medals and certificates, and we have no doubt are given to worthy and meritorious gentlemen—but just think of it. One of the most important of the arts represented at the Fair, could receive no better medal than a piece of bronze.

We hope these gentlemen will carefully preserve their medals and certifica[illegible] as mementoes of the liberality and munificence of the Proprietors of the Crystal palace.

We would here remark that we have never known the profession have other than good cause for mortification, when it has gone into any of our Fairs for exhibition. We always feel half provoked and mortified, when we have pa-

